# RNA-Seq–based transcriptomics reveals differential gene expression between two *Pisum sativum* subspecies and uncovers their molecular marker profiles

**DOI:** 10.1186/s12864-025-12419-7

**Published:** 2025-12-19

**Authors:** Kiros Tekle, Teklehaimanot Haileselassie, Kassahun Tesfaye, Kibrom B. Abreha, Haftom Brhane, Mulatu Geleta

**Affiliations:** 1 Institute of Biotechnology, Addis Ababa University, Addis Ababa, Ethiopia; 2 School of Biological Sciences and Biotechnology, Haramaya University, Haramaya, Ethiopia; 3 Bio and Emerging Technology Institute, Addis Ababa, Ethiopia; 4 Department of Plant Breeding, Swedish University of Agricultural Sciences, Lomma, Sweden

**Keywords:** Differentially expressed genes, *P. sativum* ssp. *abyssinicum*, *P. sativum* ssp. *sativum*, RNASeq, Transcriptome, Orphan crop

## Abstract

Field pea (*Pisum sativum*) holds substantial economic importance as a food and feed crop. Although *Pisum sativum* ssp. *abyssinicum* contains more protein than *Pisum sativum* ssp. *sativum*, it remains understudied and persists as an orphan crop cultivated primarily in Ethiopia and Yemen. Using RNA-Seq, gene expression patterns between these subspecies were compared, and 108,612 unigenes were identified. Off them, 69, 512 (64%) unigenes showed significant matches in major databases, yet only 2,184 matched *P. sativum* sequences, highlighting limited availability of pea genomic resources. Gene expression analysis revealed 6,704 significantly differentially expressed genes (DEGs) (3,723 down-regulated and 2,980 up-regulated in ssp. *sativum*). Most DEGs (68%) were annotated as key functional categories, including cellular processes, catalytic activity, and DNA binding. Enrichment analysis identified stress responses, defense mechanisms, and metabolic processes as prominent biological functions. Major metabolic pathways included purine metabolism, glycolysis/gluconeogenesis, and starch/sucrose metabolism. DEGs homologous to major transcription factors (bHLH, MYB, NAC, WRKY, etc.) involved in stress responses were also identified, indicating substantial transcriptional regulation differences between the two subspecies. The study also revealed the distribution of simple sequence repeats (SSRs) in the transcriptome sequences of both subspecies (10,063 in ssp. *sativum* and 11,014 in ssp. *abyssinicum)*, with trinucleotide repeats predominating, along with 4,402 polymorphic SNP markers (most showing high polymorphism information content) shared between them. These genomic resources will facilitate both the identification of genes underlying desirable traits and genomics-aided breeding, particularly benefiting the underutilized ssp. *abyssinicum*.

**Supplementary Information:**

The online version contains supplementary material available at 10.1186/s12864-025-12419-7.

## Introduction

Field Pea (*Pisum sativum* L.) belongs to the family Fabaceae and is a cool-season grain legume cultivated globally for human and livestock consumption [1–3]. It is a versatile crop utilized as a primary source of dietary protein for millions worldwide and possesses inherent resilience to environmental challenges. It is among the most protein-rich legumes and is the second most important grain legume worldwide after common bean [4]. Its ability to fix nitrogen symbiotically with rhizobium reduces the need for synthetic nitrogen fertilizers, making the crop vital for subsistence farming in marginal environments [5, 6].

In Ethiopia, two cultivated pea varieties are grown: the regular field pea (*Pisum sativum* ssp. *sativum*) and Ethiopian pea (*P. sativum* ssp. *abyssinicum*). Despite the morphological similarities, their limited cross-fertility indicates a strong reproductive barrier [7]. The origin of ssp. *abyssinicum* has long been debated due to its unique traits, narrow genetic diversity, and reproductive isolation from close relatives. Early hypotheses, such as Govorov’s 1937 (cited in [8]), proposed that subspecies *abyssinicum* arose from hybridization between *P. sativum* and *P. fulvum*, a view supported by shared traits [9]. Conversely, some studies classify it as a subspecies of *P. sativum* due to cross-compatibility and shared morphological traits, while others suggest it was independently domesticated in Ethiopia [10, 11]. However, recent genomic studies also shed more light on its taxonomic status. Phylogenetic analyses placed *P. sativum* ssp. *abyssinicum* closer to *P. sativum* ssp. *humile* than to *P. fulvum* [12–14]. Genome-wide single-nucleotide polymorphism (SNP) data confirm it clusters within *P. sativum* but as a distinct lineage, which is not in line with the hypothesis of hybrid origin [13, 15]. Although its taxonomic status is unresolved, *P. sativum* ssp. *abyssinicum* is a distinct orphan crop primarily cultivated in Ethiopia and Yemen, unlike *P. sativum* ssp. *sativum,*which is widely cultivated around the world [16, 17].. In Ethiopia, the cultivation of *P. sativum* ssp. *abyssinicum* is currently limited to specific districts in southern Tigray and northern Wollo [18]. This subspecies is notable for its protein content, tolerance to certain diseases, and value as a landrace, contributing to Ethiopia’s wealth of plant genetic resources [19].

It exhibits low genetic diversity and a highly restricted distribution, likely due to a severe bottleneck or recent origin, low adaptability from limited variability, and reproductive barriers with other pea subspecies [17]. There is a decline in the cultivation of this crop in Ethiopia due to be affected by several factors such as biotic stresses, low yields, limited land availability, and preference for more productive or commercially valuable crops, low demand due to high market prices, and limited seed supply [20, 21].

*P. sativum* ssp. *abyssinicum* is characterized by its short and climbing stature with few branches and smooth, slightly glossy seeds that range from dark violet to grey-green with violet spots and yellow cotyledons [22]. As a result of its early flowering allele (*Lf*) [22], it matures early in just 70–80 days, a relatively short lifecycle compared to other crops in the region. These traits make it particularly suited to drought-prone regions characterized by short crop growing seasons due to erratic and intermittent rainfall. In its current cultivation areas in Ethiopia, it serves as an affordable protein supplement to cereal-based diets and a source of income for farming communities as it commands premium market prices [23]. *P. sativum* ssp. *abyssinicum* is preferred for its nutritional quality, including higher crude protein, fat, and sugar content and neutral detergent fiber compared to regular field pea [18]. It is referred to as "Dero-Wot of the poor" (Chicken stew of the poor), which highlights its value as a protein source for smallholder farmers in areas where meat is not usually affordable. Thus, the crop plays a crucial role in addressing food security and malnutrition issues. Despite its significance, pea in general, and *P. sativum* ssp. *abyssinicum* in particular, lags behind other major legumes in terms of genetic characterization [24].

*P. sativum* is a diploid species, self-pollinating and with a genome size of approximately 4.26 Gb [25], which is larger than the model legume *Medicago truncatula* (~ 500 Mb) [26]) or chickpea (~ 740 Mb) [27]. The substantial disparity in genome size between field peas and related legumes is primarily attributed to the abundance of repetitive DNA elements within the pea genome, constituting over 70% of its nuclear genome [26, 28]. The estimated genome size of *P. sativum* ssp. *abyssinicum* also ranges between 4.49 GB and 4.88 GB [28, 29], which is approximately 10% larger than that of *P. sativum* ssp. *sativum*. This variation in genome size is significant among different *Pisum* species and is attributed to variations in retrotransposon insertions within their genomes [29, 30].

While *Pisum sativum* ssp. *sativum* has dominated pea genomics and breeding research, its sister subspecies *P. sativum* ssp. *abyssinicum* remains understudied, despite its distinct domestication history [13, 17]. Genomic resources for ssp. *abyssinicum* are still very limited [24]. Improved pea reference genomes now facilitate comparative genomics, enhancing genomic resources and accelerating marker-assisted breeding for traits like stress tolerance, nutritional quality, and yield. Furthermore, the development of genome-wide SNP and SSR markers in *Pisum sativum* aids in studying genetic diversity, evolution, and trait mapping [24, 31, 32].

Recent advances in cost-effective next-generation RNA sequencing technology have improved transcriptome analysis compared to previous methods like microarrays, allowing for direct sequence evaluation, detection of alternate splicing patterns, and quantification of gene expression levels [33]. Genome-wide comparative transcriptome studies have been conducted in various plant species, including field peas, with a focus on genetic marker development [24, 34–38]. However, no study has yet compared the transcriptomes of *P. sativum* ssp. *abyssinicum* and *P. sativum* ssp. *sativum,* limiting insights into their divergence in gene expression patterns and potential breeding applications. To address this gap, the present study was conducted to characterize and compare the transcriptome profiles of these two subspecies to elucidate key differences in biological processes, and develop transcriptome-derived SSR and SNP markers to expand genomic resources and tools, thereby facilitating their conservation and breeding.

## Results

### Transcriptome sequencing, quality control, and assembly

Paired-end transcriptome sequencing of *P. sativum* ssp. *sativum* and *P. sativum* ssp. *abyssinicum* resulted in 315.4 million reads, averaging 52.6 million reads per genotype (Table 1). Quality assessment of the RNA-seq datasets revealed consistent patterns within and between the two subspecies. The raw read counts ranged from 43,797,486 to 59,793,792 for *P. sativum* ssp. *sativum*, yielding an average read of 52,7870,89, and from 43,432,754 to 58,904,842 for *P. sativum* ssp. *abyssinicum*, with a mean reads of 52,343,422 indicating comparable sequencing depth between genotypes and between the two subspecies. The variance and standard deviation were 6.69e13 and 8,180,426 for *P. sativum* ssp. *sativum* (coefficient of variation: 15.5), and 6.40e13 and 7,999,100 for *P. sativum* ssp. *abyssinicum* (coefficient of variation: 15.3). the GC, Q30 and mean base quality scores also showed minimal variation within and among subspecies with an average GC content of 44.8% and a Phred score above Q30 (> 95%), indicating the suitability of the transcriptome dataset for subsequent analyses.

**Table 1:** Transcriptome summary statistics of *P. sativum* ssp. *sativum* and *P. sativum* ssp. *abyssinicum* genotypes

Parameters	*P. sativum* ssp. *sativum*			*P. sativum* ssp. *abyssinicum*			
	Ps.2770	Ps.4590	Ps.4260	Pa.2039	Pa.GL11	Pa. HW11	Mean
Raw reads	43,797,486	59,793,792	54,769,988	43,432,754	54,692,670	58,904,842	52,565,255
G + C (%)	44.9%	45.3%	44.7%	44.7%	44.6%	44.5%	44.8%
Phred score ≥ 30(%)	95.2	95.1	95.6	95.5	95.3	95.2	95.3

**Fig. 1:**
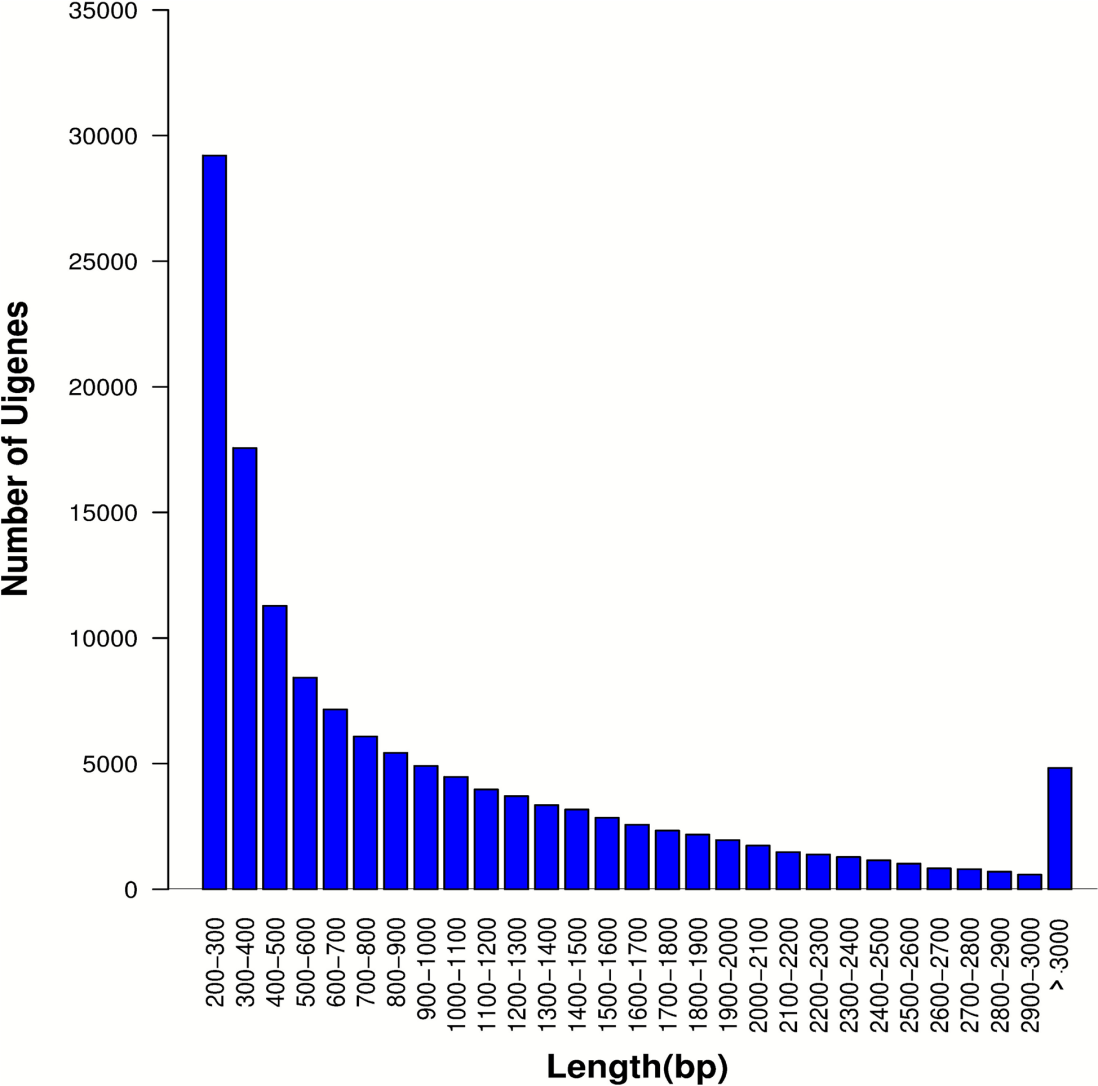
Sequence length distribution of *P. sativum* subspecies (*sativum* and *abyssinicum*) unigenes. The X-axis represents the length of the unigenes in ranges, whereas the Y- axis represents the number of unigenes in each range

After removing low-quality reads, de novo transcriptome assembly using Trinity software [39] generated 136,423 transcripts with a mean contig length of 953.82 bp and N50 of 1,516 bp with BUSCO completeness of ≥ 80% and read mapping rates of > 75%. After the removal of the redundant DNA sequences, a total of 108,612 unigenes were identified, with an average contig length of 909.28 bp and an N50 of 1,443 bp (Table 2). Among these unigenes, 48,474 (44.6%) had reads ranging from 200 to 500 bp in length, 25,644 (23.6%) ranging from 500 to 1000 bp, 23,270 (21.4%) ranging from 1,000 to 2,000 bp, and 11,224 (10.3%) exceeded 2,000 bp (Fig. 1).

**Table 2:** Transcriptome assembly summary statistics of *P. sativum* subspecies (*sativum* and *abyssinicum*)

Type	No. of contigs	Large (> = 1000 bp)	Max Length (bp)	Mean Length (bp)	N50 length (bp)	Total bases (MB)
Transcripts	136,423	46,361	13,776	953.82	1,516	130.12
Unigenes	108,612	34,494	13,776	909.28	1,443	98.76

### Functional annotation of the *P. sativum* transcriptome

BLAST searches of the 108,612 unigenes against six public databases identified significant hits (E-value ≤ 1e − 5) for 69,512 (64%) unigenes. Among these, 55,476 unigenes matched sequences in the Non-Redundant Protein (NR) database, 54,692 in UniProt, 58,496 in the Nucleotide (NT) database, and 21,988 in the Plant Transcription Factor Database (PlantTFDB). Additionally, 51,418 unigenes were annotated in the Kyoto Encyclopedia of Genes and Genomes (KEGG), and 54,692 in the Gene Ontology (GO) database (Table 3).

**Table 3:** Summary statistics of transcriptome functional annotations for two *P. sativum* subspecies across six public sequence/annotation databases

Total number of unigenes	Number of unigenes annotated in						Total number of annotated unigenes
	NR	GO	KEGG	PlantTF	UniProt	NT	
108,612	55,476	54,692	51,418	21,988	54,692	58,496	69, 512
	51.08%	50.38%	47.34%	20.24%	50.38%	53.86%	64%

Databases: *NR * non-redundant proteins, *GO* Gene Ontology, *KEGG * Kyoto Encyclopedia of Genes and Genomes, *PlantTF* Plant Transcription Factor; *UniProt* Universal Protein, *NT * Nucleotide

**Fig. 2:**
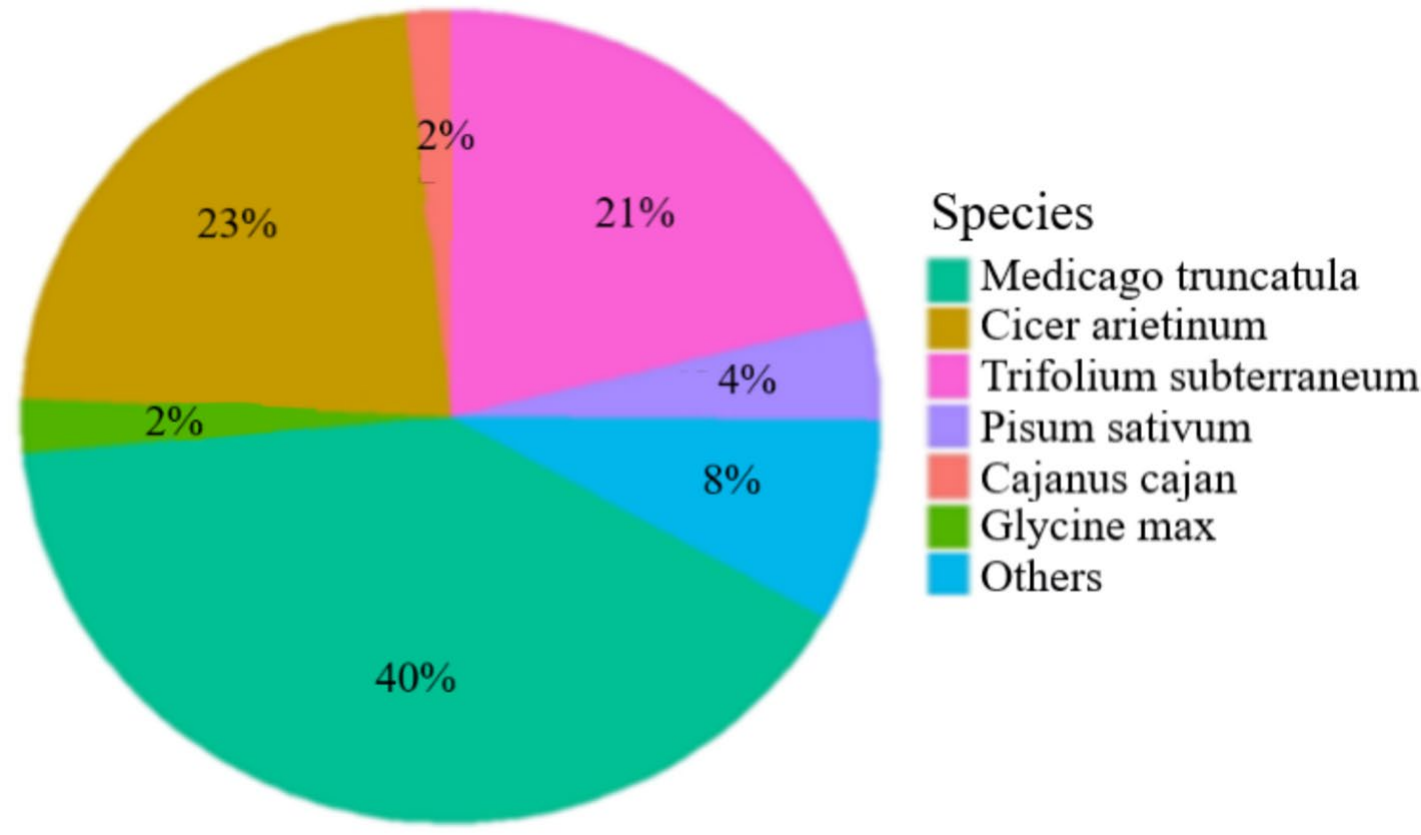
Pie chart showing the distribution of BLAST hits for 55,476 unigenes from the two *P. sativum* subspecies against the NCBI non-redundant Protein (NR) database

About 21,896 (39.5%) of the 55,476 unigenes annotated in the non-redundant protein database (NR) showed the highest similarity to sequences from *Medicago truncatula*. The next top hits were with *Cicer arietinum*, comprising 12,622 (22.8%) unigenes, followed by *Trifolium subterraneum* with 11,854 (21.4%) unigenes, and only 2,184 (3.94%) found hits in *P. sativum* sequences (Fig. 2).

**Fig. 3:**
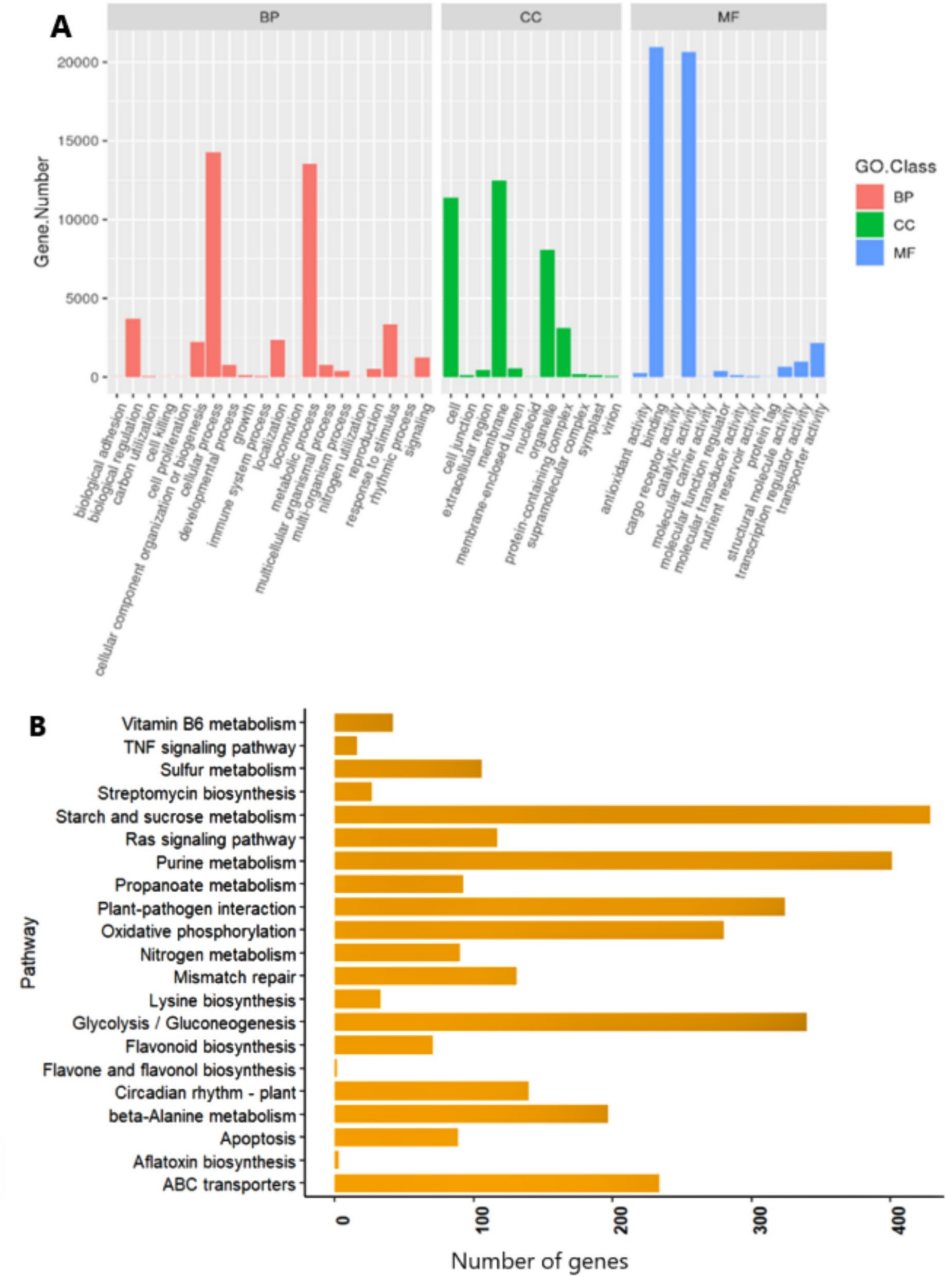
**A** Gene ontology (GO) annotation of *P. sativum* unigenes across biological process (BP), cellular component (CC), and molecular function (MF) categories. The X-axis represents GO terms, and the Y-axis shows the number of unigenes per term; (**B**) Distribution of *P. sativum* unigenes across different Kyoto Encyclopedia of Genes and Genomes (KEGG) annotations pathway categories. X-axis = the number of unigenes; Y-axis = KEGG sub-pathways

Gene Ontology (GO) annotation analysis identified a total of 43 GO terms distributed among the three primary GO categories: 20 terms in Biological Process (BP), 11 terms in Cellular Component (CC), and 12 terms in Molecular Function (MF). Within the class of biological processes GO-term, the cellular processes (14,268 unigenes), metabolic processes (13,530 unigenes), and biological regulation (3,697 unigenes) are the most abundant (Fig. 3). Proteins associated with the membrane (12,484), cell (11,391), and organelle (8,047) unigenes are the top terms in the cellular component class. Among the most abundant terms in the molecular function class, catalytic (20,938), binding (20,657), and transport activity (2,159) unigenes were assigned to these terms (Fig. 3A).

The unigenes were also functionally annotated using the Kyoto Encyclopedia of Genes and Genomes (KEGG) database to predict putative roles based on their sequence homology. Of the 54,692 unigenes with significant BLAST hits (*e*-value cutoff = 1e^−5^) in the KEGG database, 24,294 (44.4%) unigenes were classified under five major KEGG classes: Metabolism (11,337 unigenes), genetic information processing (4,231 unigenes), cellular processes (3,377 unigenes), environmental information processing (3,776 unigenes), and organismal systems (464 unigenes). These unigenes were further assigned to 196 pathways, with the most prominent being Starch and sucrose metabolism (429), purine metabolism (401), glycolysis/gluconeogenesis (340), plant-pathogen interaction (324), oxidative phosphorylation (280), and ABC transporter (234) (Fig. 3B).

BLAST searches against the Plant Transcription Factor Database (PlantTFDB) identified significant matches (P-value ≤ 1e⁻^5^) for 21,988 unigenes. Among the 62 protein families found, the bHLH protein family was the most abundant (2,146 unigenes), followed by MYB-related (1,600), NAC (1,518), ERF (1,187), and C2H2 family proteins (1,110) related unigenes (Supplementary Fig. 1a). Among the 160 plant species showing at least one significant match, *Medicago truncatula, Trifolium pretense*, and *Malus domestica* exhibited the highest representation of transcription factor sequence matches (Supplementary Fig. 1b).

### Transcriptomic relationships between *P. sativum* ssp. *sativum *and *P. sativum* ssp. *abyssinicum*

**Fig. 4:**
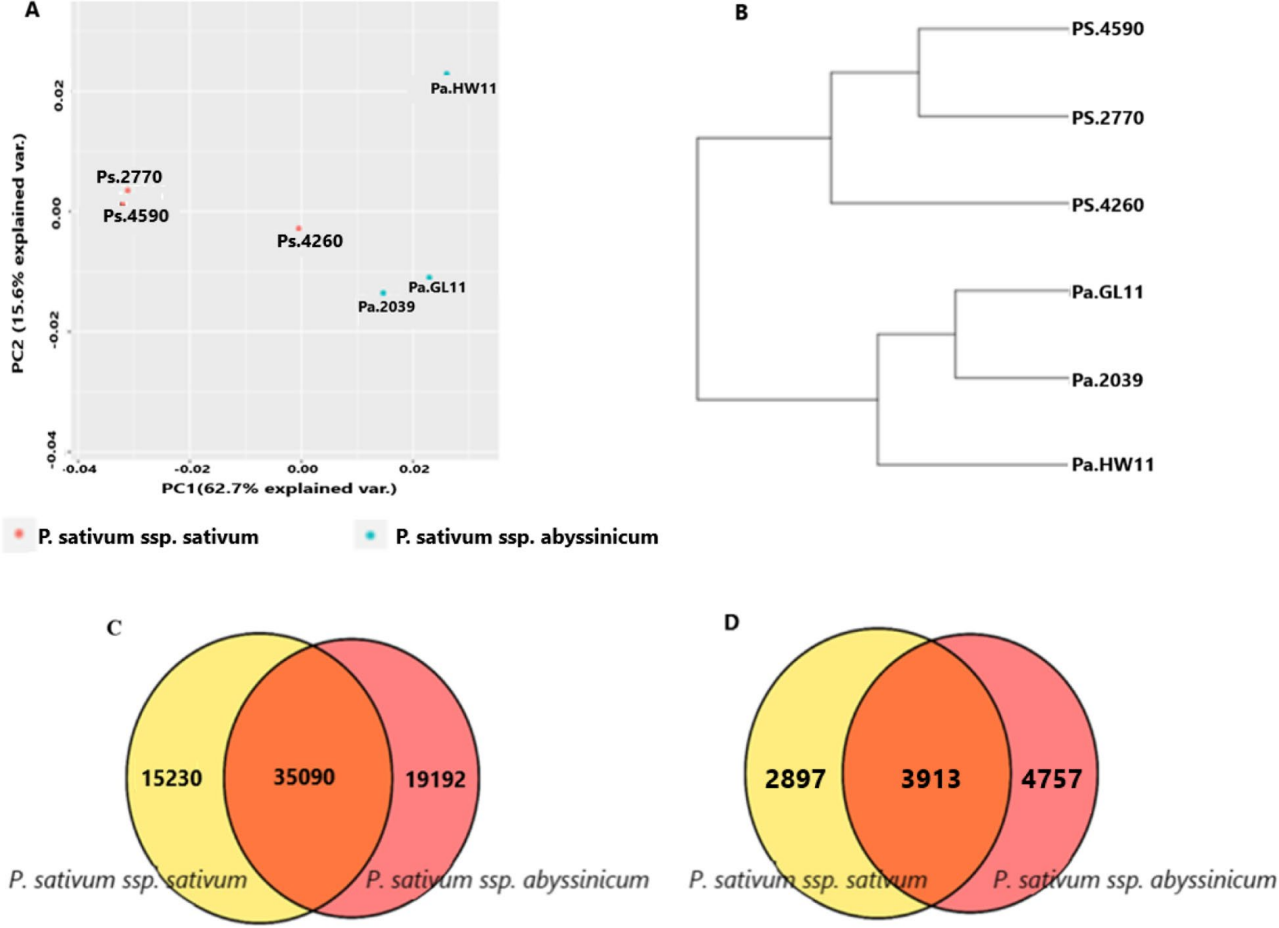
Gene expression-based analysis depicting relationships between the two *P. sativum* subspecies: (**A**) PCA scatter plot, (**B**) Unweighted pair group method with arithmetic mean (UPGMA) dendrogram, (**C**), Venn diagram of shared and subspecies-specific expression of unigenes, and (**D**). Venn diagram depicting the distribution of the significantlly differentially expressed unigenes among the two subspecies with the adjusted *p*-value < 0.01 and log2FC > 2

Gene expression patterns were analyzed using principal component analysis (PCA) to examine the relationship between the six genotypes representing *P. sativum* ssp. *sativum* and *P. sativum* ssp. *abyssinicum*. The first and second principal components (PC1 and PC2) accounted for 62.7% and 15.6% of the total variation, respectively. PC1 clearly distinguished the two subspecies, while both components revealed variation within subspecies (Fig. 4A). Euclidean distance-based clustering of the transcriptome profiles showed distinct grouping with the three *P. sativum* ssp. *sativum* genotypes (PS.4590, PS.2270, and PS.4260) formed one clade, separate from the three *P. sativum* ssp. *abyssinicum* genotypes (Pa.GL11, Pa.2039, and Pa. HW11) (Fig. 4B). Both analyses demonstrated clear differences in gene expression patterns between the two subspecies.

Of the total of 108,612 unigenes identified, 69,512 unigenes were annotated in the major databases using an E-value threshold of ≤ 1e − 5. Among these unigenes, 35,090 (50.5%) expressed unigenes were shared by both species, while 19,192 (27.6%) and 15,230 (21.9%) unigenes were uniquely detected in *P. sativum* ssp. *abyssinicum* and *P. sativum* ssp. *sativum*, respectively (Fig. 4C, Supplementary Table 1).

### Differentially expressed genes (DEGs) and cluster analysis

**Fig. 5:**
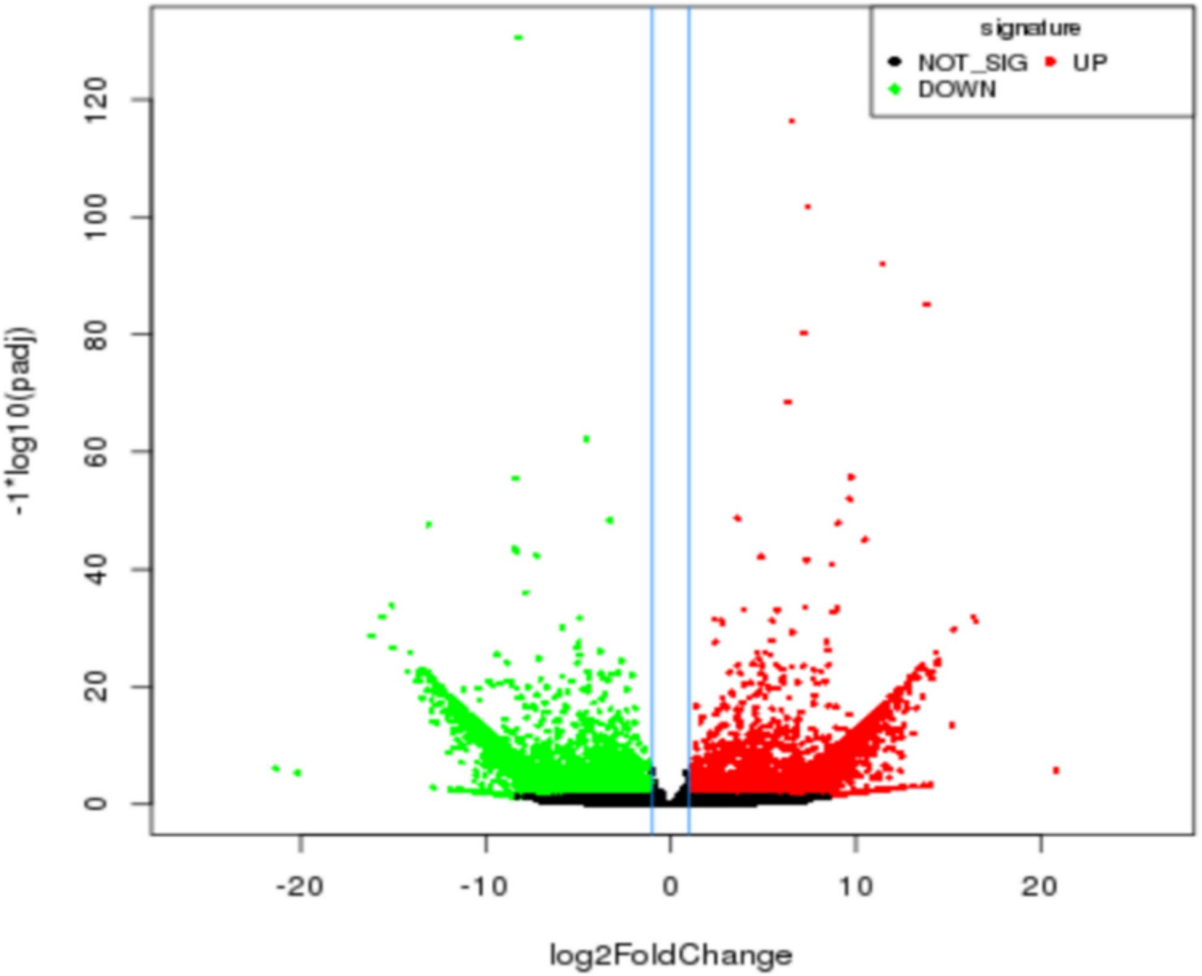
Volcano plots of significantly differentially expressed genes between the two *P. sativum* subspecies. Each dot represents a gene. Red dots and green dots represent upregulated genes in *P. sativum* ssp. *sativum* and *P. sativum* ssp. *abyssinicum*, respectively. Black dots represent genes that were not significantly differentially expressed (Not sig) between these subspecies

Applying a strict filtering thresholds of *p*-value < 0.01 and Log2 fold-change > 2 to the annotated unigenes, 11,567 significantly differentially expressed genes were obtained between the two *Pisum sativum* subspecies. Among these, 4,764 (41.2%) were significantly upregulated, while the other 6,803 (58.8%) were significantly downregulated in *P. sativum* ssp. *sativum* genotypes (Fig. 5; Supplementary Table 2). Gene expression analysis of the significant DEGs also shows the relationship between the two Pisum sub-species, in which 3,913 (33.8%) expressed unigenes were shared by both species, while 2,897 (25.1%) and 4,757 (41.1%) unigenes were uniquely detected in *P*. *sativum* ssp. *Sativum and P. sativum* ssp. *abyssinicum*, respectively (Fig. 4D).

**Fig. 6:**
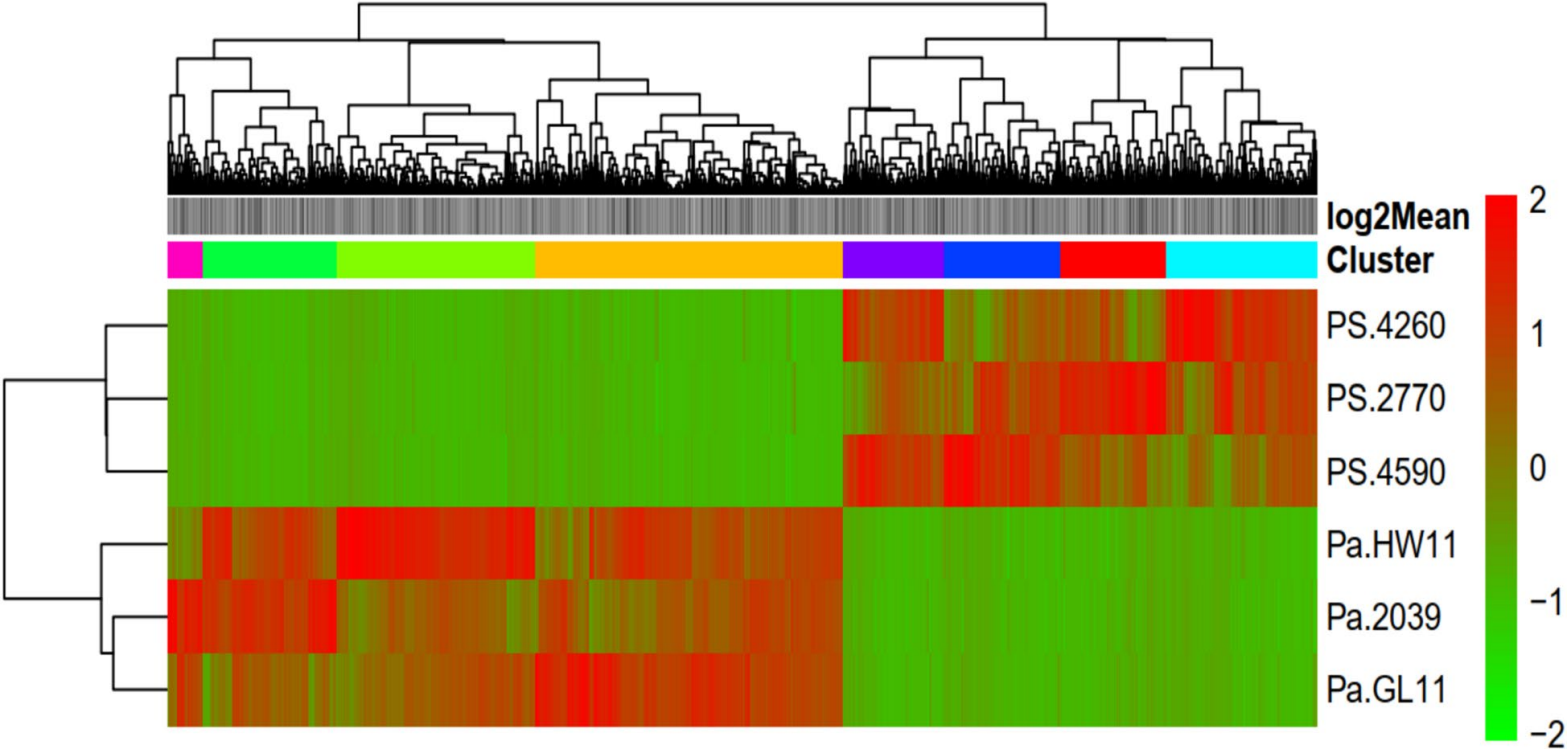
Heatmap of two-way hierarchical clustering analysis of significantly differentially expressed genes across six genotypes of the two *P. sativum* subspecies. Rows represent genotypes (forming two clusters representing the two subspecies), and columns represent genes clustered into eight distinct expression profiles (indicated by colored bars above the heatmap). Red and green denote upregulation and downregulation, respectively, relative to the comparison between the subspecies

Hierarchical clustering of the 11,567 significant DEGs (Log2FC > 2) showed similar gene expression patterns among genotypes of each subspecies but different patterns between the subspecies. Among the resulting eight clusters, the first four clusters represent downregulated genes and the remaining four clusters represent upregulated genes in *P. sativum* ssp. *abyssinicum* genotypes, compared to *P. sativum* ssp. *sativum* genotypes. The similarity in expression pattern is more evident in downregulated genes than in upregulated genes in both subspecies (Fig. 6).

### Annotation of differentially expressed genes (DEGs)

Of the 11,567 significantly differentially expressed unigenes (DEGs), 6,704 (58%) were functionally annotated in at least one of the six major databases (Supplementary Table 2). The majority of annotated DEGs were assigned Gene Ontology (GO) terms (4,562), followed by KEGG (1,481) and PlantTFDB (2,478) annotations. Additionally, the DEGs had strong representation in the reference databases: NR (6,091), NT (6,036), and UniProt (6,023) databases.

**Fig. 7:**
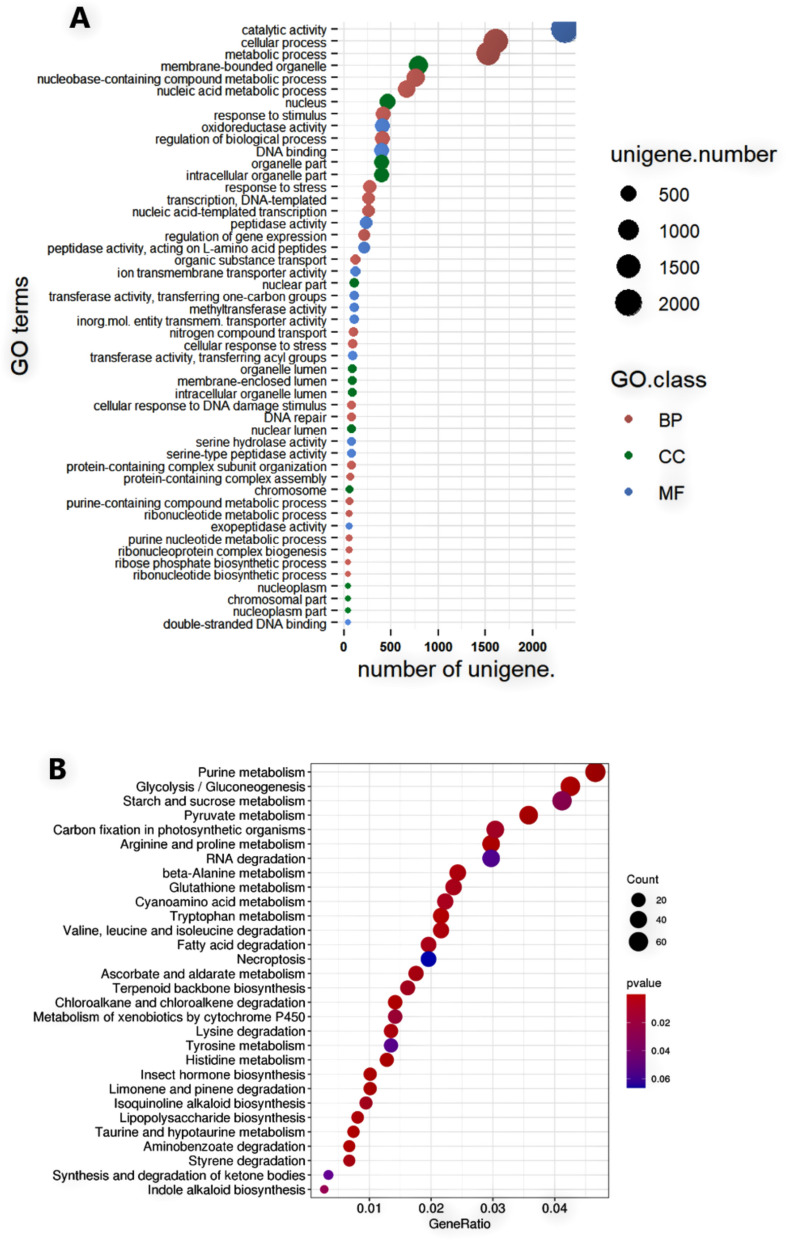
**A** Bubble plot of significantly differentially expressed genes (DEGs) enriched across Gene Ontology (GO) classes: biological processes (BP), molecular functions (MF), and cellular components (CC). Y-axis = Enriched GO terms; X-axis: Number of DEGs per term. Bubble size represents gene count; (**B**) Bubble plot displaying enrichment of significantly differentially expressed genes (DEGs) in KEGG pathways using. Y-axis = pathway names; X-axis = number of DEGs per pathway. Bubble size represents enrichment ratio while colors represent *p*-values

In the case of Gene Ontology annotations, most of the significantly enriched DEGs (padj < 0.05) were annotated under the biological process (BP) class, particularly cellular process (GO:0009987; 1,608 genes), metabolic process (GO:0008152; 1,532 genes), organic substance metabolic process (GO:0071704; 1,397 genes), primary metabolic process (GO:0044238; 1,302 genes), and cellular metabolic process (GO:0044237; 1,273 genes). In the cellular component class, membrane-bounded organelle (GO: 0043227; 796 genes), nucleus (GO:0005634; 465 genes), intracellular organelle part (GO: 0044446; 398 genes), and organelle part (GO: 0044422; 398 genes) were the top four most frequent GO terms. Catalytic activity (GO:0003824; 2345 genes), oxidoreductase activity (GO: 0016491; 404 genes), DNA binding (GO: 0003677; 401 genes), and peptidase activity (GO:0008233; 234 genes) were also among the prominent GO terms in the molecular function class (Fig. 7A; Supplementary Table S3).

The 1,359 upregulated and 122 downregulated DEGs were assigned to 183 pathways spanning 23 functional classes (Supplementary Table S4). The most abundant categories are signal transduction (29 pathways), Carbohydrate metabolism (15 pathways), Lipid metabolism (15 pathways), and amino acid metabolism (14 pathways). The three most abundant metabolic pathways associated with significant DEGs were purine metabolism (ko00230; 69 genes), Plant hormone signal transduction (ko04075; 66 genes), Spliceosome (ko03040: 65 genes), glycolysis/gluconeogenesis (ko00010; 62 genes), and starch and sucrose metabolism (ko00500; 61 genes). Within the Environmental Information Processing class, the NF-kappa B signaling pathway (ko04064:42 genes), MAPK signaling pathway (ko04016; 37 genes), and signal transduction and ABC transporters (ko02010; 24 genes) were the most abundant. Among the pathways within Cellular Processes, peroxisome (ko04146), endocytosis (ko04144), and autophagy (ko04140), were the top three with 31, 30, and 23 genes, respectively. Within Genetic Information Processing, RNA transport (ko03013; 47 genes), RNA degradation (ko03013; 44 genes), and ribosome (ko03010; 40 genes) were the top three. Within the Environmental Adaptation pathway class, the DEGs were assigned only to plant-pathogen interaction (ko04626: 40 genes) and circadian rhythm-plant (ko04712; 19 genes). (Fig. 7B; Supplementary Table S4).

A BLAST search against the transcription factor (TF) database (E-value < 1e-5) identified 2,478 (21.4%) significant DEGs belonging to 56 TF protein families. The most abundant families were bHLH-related **(**244 DEGs), MYB (217 DEGs), and NAC (144 DEGs), followed by ERF (138 DEGs), B3 (131 DEGs), C2C2 (119 DEGs), and C3H (103 DEGs). These DEGS showed homology to proteins from 134 plant species, with the highest matches being those of *Medicago truncatula, Trifolium pratense*, and *Malus domestica*, with 154, 142, and 135 DEGs, respectively (Supplementary Table 5).

### 3D protein structure prediction

**Fig. 8:**
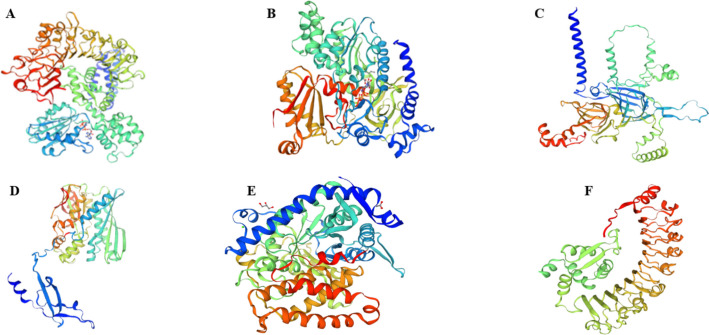
Predicted 3D protein structures of six unigenes. The 3D structures include three downregulated unigenes: (**A**) DN33997_c1_g2_i5, a disease-resistance protein RPP13 of the TIR-NBS-LRR class; (**B**) DN32002_c0_g1_i7, a Jasmonate-L-amino acid synthetase JAR4; (**C**) DN29106_c0_g1_i1, a legumin J and K storage protein; and six upregulated unigenes: (**D**) DN32208_c0_g1_i4, a glutamate receptor 3.6-like (GLR3.6-like); (**E**) DN22559_c0_g1_i2, a malate synthase located in glyoxysomal; and (**F**) DN33714_c3_g5_i5, a probable disease resistance protein similar to At5g66900

Computational homology modeling was employed via the SWISS-MODEL (expasy.org) platform to predict the tertiary structures of selected differentially expressed genes base on their log2FC change and assess their functional implications. Among the downregulated genes in *P. sativum* ssp. *sativum*, DN33997_c1_g2_i5 exhibited 80.0% sequence identity with the disease-resistance protein RPP13 of the TIR-NBS-LRR class protein family. Notably, DN32002_c0_g1_i7 displayed a 97.0% structural similarity to Jasmonate-L-amino acid synthetase JAR4, while DN29106_c0_g1_i1 showed 94.0% similarity to legumin-K and J storage proteins (Fig. 8A-C).

Tertiary structure predictions for the upregulated genes revealed that DN32208_c0_g1_i4, DN22559_c0_g1_i2, and DN33714_c3_g5_i5 exhibited high-confidence sequence homology > 98%) with glutamate receptor 3.6-like (GLR3.6-like), malate synthase (glyoxysomal), and a probable disease resistance protein At5g66900 (Fig. 8D-F). These high-confidence structural predictions were achieved through template-based modeling utilizing Swiss-Model [40], ensuring reliable insights into protein functionality.

### The distribution of SSRs in the unigenes

The *P. sativum* ssp. *sativum* (73,106) and *P. sativum* ssp. *abyssinicum* (79,085) unigenes were examined for SSRs using the Microsatellite (MISA) software. A total of 11,685 SSRs were identified from 9,441 unigenes in *P. sativum* ssp. *abyssinicum*, with 1,304 unigenes containing more than one SSR. Among these SSRs, 11,015 were classified as perfect SSRs, while 570 were compound SSRs (Supplementary Table 6 A). The distribution of perfect SSRs revealed di (19%), tri (31.4%), tetra (0.7%), penta (0.21%), and hexanucleotide (0.49%) repeats in *P. sativum* ssp*. abyssinicum* (Supplementary Fig. 2 A). In *P. sativum* ssp. *sativum*, 10,624 SSRs were identified from 8,580 unigenes, with 1,241 unigenes containing more than one SSR. Of these, 10,046 were perfect SSRs and 561 were compound SSRs (Supplementary Table 6B). The distribution in *P. sativum* ssp. *sativum* was di (20.3%), tri (31.5%), tetra (0.81%), Penta (0.45%), and hexanucleotide (0.54%) (Supplementary Fig. 2 A).

The mononucleotide SSR repeat motifs A/T constituted 99.1% and 99.5%, whereas G/C motifs constituted 0.86% and 0.47% in *P. sativum* ssp. *abyssinicum* and *P. sativum* ssp. *sativum*, respectively. Among the di-nucleotide repeats, AG/CT, AT/AT, and AC/GT motifs were dominant, while CG/CG repeats were less common in both species, with a percentage of 72.21%, 11.96%, 10.74%, and 0.9 in *P. sativum* ssp. *abyssinicum* and 75.1%, 12.5%, 12.3% and 0.15 in *P. sativum* ssp. *sativum*. Tri-nucleotide SSR repeats AAG/CTT, ATC/GAT, and AAC/GTT were found to be the most common motifs, with compositions of 28.27%, 18.48%, 16.71%, and 26.5%, 19.2%, 16.9% in *P. sativum* ssp. *abyssinicum* and *P. sativum* ssp. *sativum,* respectively. On the other hand, the CCG/CGG and ACG/CGT repeats were less frequent, 0.52% and 1.15% in *P. sativum* ssp. *abyssinicum* and 0.51% and 1.3% in *P. sativum* ssp. *sativum*. Furthermore, analysis of Tetra-nucleotide SSR repeats showed AAAT/ATTT as the most prevalent motif in *P. sativum* ssp. *abyssinicum* (45.45%) and *P. sativum* ssp. *sativum* (29.6%)*.* The diversity of penta and hexanucleotide SSR repeats was observed with varying numbers in both species (Supplementary Fig. 2B).

The frequency of SSR repeats decreased as the number of repeats increased. For instance, the number of mononucleotide repeats with a repeat of 10, 11, and 12 were 2,611, 959, and 454 in *P. sativum* ssp. *sativum,* respectively. Similarly, there were 2, 985, 983, and 564 SSRs in *Pisum abyssinicum*. The number of dinucleotide SSRs with repeats of six and seven were 769 and 453 in *P. sativum* ssp. *sativum,* and 764 and 463 in *P. sativum* ssp. *abyssinicum,* respectively. Trinucleotide SSRs with a repeat length of five were found to be more than twice as common as those with a repeat length of six. Specifically, there were 1,815 and 2,041 SSRs with five repeats in *P. sativum* ssp. *sativum* and *P. sativum* ssp. *abyssinicum*, respectively, compared to 717 and 769 SSRs with six repeats in these species.

The examination of SSRs was also conducted on 108,612 unigenes shared by both species using the online tool MISA. Out of these, 12,749 unigenes contained a total of 15,110 SSRs. Among the identified SSRs, 14,154 were regular SSRs, and 956 were compound SSRs (Supplementary Fig. 2 A).

### SNP markers, cluster, and principal coordinate analyses

SNP calling for each genotype using de novo assembled unigenes as references resulted in the number of SNPs ranging from 157,576 (in Pa.2039) to 243,489 (in Ps.4260). Merging and filtering these SNPs resulted in 97,241 SNPs shared among the three *P. sativum* ssp. *abyssinicum* genotypes, 88,649 SNPs shared among the three *P. sativum* ssp. *sativum* genotypes, and 4,402 SNPs shared among all six genotypes (Supplementary Fig. 3; Supplementary Table 7). Of these shared SNPs across the six genotypes, bi-allelic and tri-allelic SNPs 4,360 (99.05%) and 42 (0.95%), respectively. Most SNPs (57.2%) were highly informative with PIC values ≥ 0.30.

**Fig. 9:**
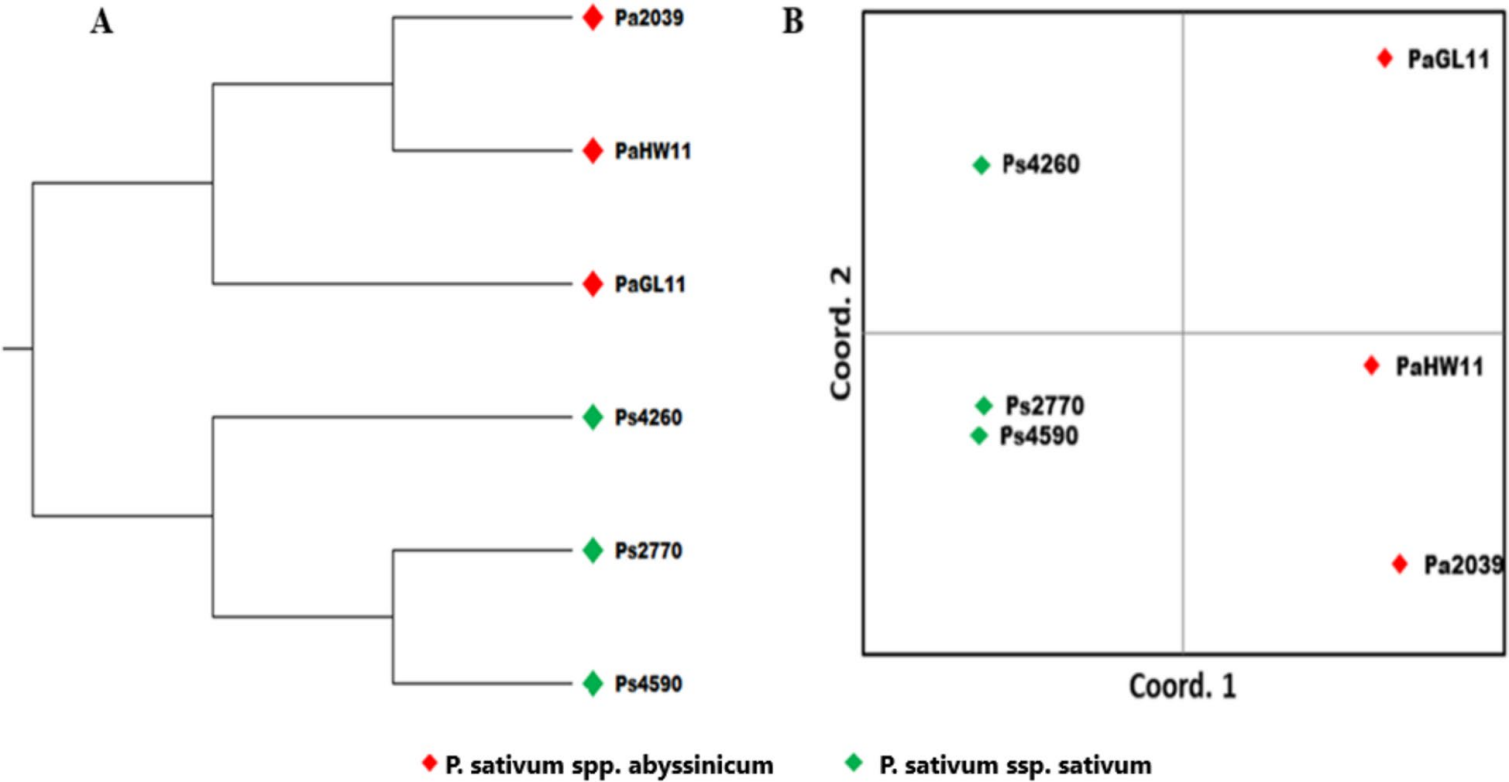
Genetic relationships between six *P. sativum* genotypes (*P. sativum* ssp. *sativum* and *P. sativum* ssp. *abyssinicum*) visualized by (**A**) Unweighted pair group method with arithmetic mean (UPGMA) clustering and (**B**) principal coordinate analysis (PCoA) using 4,402 shared SNPs

Analysis of the 4,402 shared SNP markers revealed a clear genetic separation of the six genotypes into their respective subspecies as determined by both UPGMA cluster analysis and principal coordinate analysis (PCoA) (Fig. 9). In PCoA, the first principal coordinate accounted for 89.7% of the observed total genetic variation. The pairwise genetic distance between the six samples ranged from 0.2 to 0.04. Pairwise genetic distances between genotypes ranged from 0.04 (between *P. sativum* ssp. *sativum* genotypes; PS.2270 and PS.4590) to 0.2 (between genotypes of the two subspecies; Pa.2039 and PS.4260) (Supplementary Fig. 3 A and B).

## Discussion

Field peas play a crucial role in agriculture by providing food, feed for livestock, and sustainable nitrogen inputs, with significant potential for future food systems and plant-based food industries [41, 42]. However, in *P. sativum* ssp. *abyssinicum*, the scarcity of genomic resources and limited understanding of its biological processes and evolutionary relationships with *P. sativum* ssp. *sativum* hinders the development and application of novel breeding tools for its improvement. Over the past decades, advances in next-generation sequencing and bioinformatics analysis tools, along with increased efficiency and reduced costs, have revolutionized RNA sequencing, making it more accessible for research across diverse plant species. RNA-Seq is a powerful tool for gaining critical insights into genetic regulation, deciphering molecular mechanisms of plant responses to biotic and abiotic stresses, and facilitating the development and application of molecular markers in breeding programs. Despite these advances, no transcriptome-based study has aimed to unravel the key pathways and evolutionary dynamics between *P. sativum* ssp. *sativum* and *P. sativum* ssp. *abyssinicum* has been reported so far.

The present study aimed to shed light on this gap by generating high-quality transcriptome data via high-throughput RNA-seq to analyze transcriptome profiles, identify differentially expressed genes (DEGs), and explore key pathways shaping the evolutionary relationship between these subspecies. We also characterized the distribution of SSRs in the transcriptomes of the two subspecies and identified SNP markers, which hold significant potential for advancing genomic tools and advanced breeding methods to enhance these crops and develop elite cultivars. Because each sequenced plant represented a distinct genotype, the study lacked true biological replicates, and the differential-expression results should therefore be viewed as exploratory. Nonetheless, the patterns observed provide a useful foundation for future work. Follow-up studies incorporating biological replicates for each genotype will be essential to validate these findings and enable more robust statistical testing.

De novo RNA-Seq assembly and analysis of all genotypes form the two subspecies' (*P. sativum* ssp. *sativum* and *P. sativum* ssp. *abyssinicum)*yielded 108, 612 unigenes of which 69, 512 (64%) were annotated against public databases (Nt, Nr, UniProt, KEGG, GO and TF). Annotation percentages vary across pea and other legume transcriptomes, with some studies reporting lower values [38], 43% and 47% in two related pea cultivars (Kaspa and Parafield) respetevely while others report higher rates [34], (74% and 79%) annotated against UniProt and *Medicago truncatula* predicted proteins database with e-value lower than 1e-5 in pea species, reflecting differences in material used, sequencing depth, assembler performance, and annotation pipelines. Overall, our results are consistent with previous pea transcriptomes, providing a robust basis for downstream functional analyses, while the unannotated fraction may contain novel or species-specific transcripts.

The assembly metrics also revealed longer contig and transcript lengths compared to earlier field pea studies [34–36], with unigene counts comparable with the 77,273 unigenes reported by [43]. Among the 55,476 unigenes annotated in the non-redundant protein database (NR), only 2,184 (3.94%) unigenes showed homology to *P. sativum* sequences, highlighting the limited genomic resources available for *Pisum* species [35, 44, 45]. This may also be partly due to undetected short reads during BLAST matches [46]. Notably, a high proportion of unigenes aligned with *Medicago truncatula*, consistent with its role as a legume model species [47]. Altogether, the high-quality transcriptome data generated in this study provide valuable resources for genomic research on these subspecies.

To gain insights into shared molecular characteristics, functional annotation of 108,612 unigenes assembled from the two subspecies was performed. Analysis of these shared unigenes revealed predominant Gene Ontology (GO) terms, KEGG pathways, and transcription factor families, highlighting conserved biological processes and molecular functions between the subspecies*.* Key metabolic pathways identified included signal transduction, carbohydrate metabolism, and amino acid metabolism [38, 48], consistent with the known complexity of legume metabolism. The most frequent transcription factors (such as bHLH, MYB, and NAC) shared by *P. sativum* ssp. *sativum* and *P. sativum* ssp. *abysinicum* also represent a large proportion of genes in pea and other crops [49–52]. This comparative analysis of shared unigenes provides critical insights into the conserved genetic architecture across the subspecies, facilitating future trait improvement in *P. sativum* ssp. *abysinicum*.

UPGMA clustering analysis of the transcriptome profiles revealed that the three genotypes of *P. sativum* ssp. *sativum* (PS.4590, PS.2270, and PS.4260) clustered together in a distinct clade separate from the three *P. sativum* ssp. *abyssinicum* genotypes (Pa2039, PaGL11, and PaHW11). The result indicates a clear transcriptome divergence between the two subspecies. However, the absence of pronounced clustering in the PCA analysis suggests a close evolutionary relationship. Hierarchical clustering of the DEGs showed a subspecies-specific expression pattern, consistent with the influence of speciation on the biological processes, as observed in other crops [53]. Notably, the two subspecies exhibit clear differences in morphology, growth and development, yield potential, flowering time, protein content, and palatability [8, 16, 54–56]. These phenotypic differences likely explain the substantial number of DEGs associated with catalytic activity, cellular processes, and metabolic processes, as indicated by GO terms. The top three pathways associated with the DEGs were Purine metabolism (ko00230: 69 genes), Glycolysis/Gluconeogenesis (ko00010: 63 genes), and starch and sucrose metabolism (ko00500; 61 genes).

The significant DEGs identified in this study included genes from 56 TF families, with bHLH, MYB, and NAC being the most abundant. These TFs are known to regulate plant stress responses [57]. Among DEGs encoding these TFs, DN33997_c1_g2_i5, DN32002_c0_g1_i7, and DN29106_c0_g1_i1 were down-regulated, while DN32208_c0_g1_i4, DN33714_c3_g5_i5 and DN22559_c0_g1_i2 were up-regulated in *P. sativum* ssp. *sativum*. These differences in gene expression patterns highlight the molecular divergence between the two subspecies. Molecular differences show the interspecific disparities between the two field pea species, further supporting the documented differences in agronomic traits, such as seed morphology, phenology, and physiological attributes, as elucidated by [8].

Three-dimensional (3D) protein structures were predicted for several differentially expressed genes*.* Among the downregulated genes in *P. sativum* ssp. *sativum*, DN33997_c1_g2_i5 shared over 80% sequence homology with the gene encoding the RPP13-like disease-resistance protein, which is implicated in diverse stress responses across species. The gene confers resistance to downy mildew in Arabidopsis [58], abscisic acid-mediated heat stress resistance in maize [59], abiotic stress tolerance in barley [60], and powdery mildew resistance in wheat [61]. Another downregulated unigene in *P. sativum* ssp. *sativum*, DN32002_c0_g1_i7 showed high sequence similarity to a gene encoding Jasmonate-L-amino acid synthetase JAR4, a key enzyme in plant defense signaling pathways, indicating its involvement in herbivore defense mechanisms [62]. Furthermore, the unigene DN29106_c0_g1_i1 exhibited over 94% sequence homology with the Legumin-K and -J genes, evolutionarily conserved genes encoding storage proteins crucial for nitrogen allocation in pea seeds. These isoforms possess structural features that influence nutrient dynamics during development and industrial applications [63].

Among the upregulated genes in *P. sativum* ssp. *sativum*, DN32208_c0_g1_i4 showed a 99% similarity to the glutamate receptor 3.6-like (GLR3.6-like) gene, which plays a vital role in plant signaling and defense against environmental stresses and insect attacks [64, 65]. The unigene DN22559_c0_g1_i2 shares over 98% homology with a gene encoding glyoxysomal malate synthase, a key enzyme for maintaining malate balance during plant growth and development. It also contributes to the regulation of photosynthesis and facilitates malate transport via ALMT proteins [66, 67]. Similarly, the unigene DN33714_c3_g5_i5 demonstrated over 99% sequence homology with the gene probably encoding disease-resistance protein At5g66900, which plays a significant role in immunity against various pathogens in Arabidopsis [68, 69].

The quality control assessment of the raw reads revealed 79,085 in *P. sativum* ssp. *abyssinicum* and 73,106 unigenes in *P. sativum* ssp. *sativum* with an average G + C content of 44.8%. G + C-rich regions of DNA are usually transcriptionally active regions, facilitate complex gene regulation, and influence genome organization [70, 71]. The genomic G + C content also varies widely among different species and genomic regions, with the grass family exhibiting higher G + C levels [71]. Studies indicated that the differences in G + C content are associated with environmental adaptation and evolutionary significance [71–73]. In general, monocots have higher overall genomic G + C content than dicots. In dicots, the G + C content within genes is much higher than in non-genic regions, ranging from 43.4% to 44.5% [74]. Hence, the G + C content of 44.8% obtained in the present study for the *P. sativum* ssp. *sativum* and *P. sativum* ssp. *abyssinicum* unigenes is in line with findings reported for other dicots.

The SSR sequences are distributed throughout the genome and are highly polymorphic due to variations in the number of repeats. SSR mutations primarily arise from DNA polymerase slippage during replication and errors in DNA repair processes, with mutation rates further influenced by the motif’s type, length, and sequence purity [75, 76]. SSR markers have been widely used in genetic mapping, marker-assisted selection, and genetic diversity studies and play a significant role in crop improvement programs, germplasm conservation, and understanding the genetic basis of important traits in crops [77–80]. The distribution, density, and types of SSRs, along with the nucleotide composition of their repeat motifs, also vary both among genomes of different species and regions within the same genome [81–86]. In this study, mononucleotide SSRs are found to be the most dominant repeat types and cover more than half of the total SSR repeat motifs. A/T, AT/TA, and AAG/CTT repeats are also much more common than their G/C, CG/GC, and CCG/CGG counterparts, respectively. This dominance pattern of repeat motives is similar to reports for various legume crops such as faba bean, rice bean, alfalfa, and cowpea, *Medicago sativa* highlighting a widespread trend of those SSR types across legume species [87–92]. A/T-rich SSR motifs, such as A/T, AG/CT, and AAG/CTT, are abundant in plant genomes due to their higher susceptibility to replication slippage and weaker purifying selection, whereas the evolutionary decline of GC-rich motifs like CG/GC and CCG/CGG reflects their greater stability and stronger selective constraints, particularly in coding regions[83, 93]. The G + C content of field pea unigenes (44.8%) exceeds that of the SSRs identified from these unigenes (0.8%). These findings align with previous research in various dicot species [87, 88, 90–92, 94].

Among the 324,346 SNPs identified, 91,678 were unique to *P. sativum* ssp. *abyssinicum* and 77,105 SNPs were unique to *P. sativum* ssp. *sativum*. Clustering analysis using 4,402 shared SNP markers further confirmed the genetic divergence between the two subspecies. This finding aligns with previous studies using SSR and SNP markers, which consistently demonstrate that *P. sativum* ssp. *abyssinicum* is genetically distinct and forms a separate cluster from other *Pisum* species [13, 17, 32, 95–99]. The transcriptome-based markers developed in this study will serve as valuable molecular tools to advance genomic research and genomics-driven breeding in these two crops. The newly developed SNP markers can enhance breeding programs by enabling genetic diversity analysis, trait mapping, and marker-assisted selection, ultimately accelerating the development of improved field pea varieties. Additionally, they provide insights into population structure, evolutionary relationships, and intraspecies genetic diversity, supporting conservation efforts and sustainable crop management strategies.

## Materials and methods

### Plant materials and growth conditions

Three genotypes of *P. sativum* ssp. *sativum* including Ps2770, Ps4260, and Ps4590, and three genotypes of *P. sativum* ssp. *abyssinicum* including Pa2039, PaGL11, and PaHW11, were used in this study. Ps2770, Ps4260, Ps4590, and Pa2039 were randomly sampled from accessions previously used by [24], while PaGL11 and PaHW11 were sampled from farmers’ fields in North Wello and Southern Tigray, respectively, in Ethiopia to represent the genetic diversity and geographic variation within *P. sativum* ssp. *sativum* and ssp. *abyssinicum*. The three *P. sativum* ssp. *sativum* and three *P. sativum* ssp. *abyssinicum* samples each represent distinct genotypes rather than biological replicates. Thus, these samples capture inter-genotype variation within species rather than the within-genotype biological variance. Accordingly, we treat the present study as an exploratory, genotype-diverse comparison of transcriptional states between species. The genotypes were grown in 1.5 L plastic pots in a greenhouse at the Swedish University of Agricultural Sciences (SLU), Alnarp, Sweden. The greenhouse chamber was set to a 16-h photoperiod, with day/night temperatures of 21/18 °C, a light intensity of 200 µmol m⁻^2^ s⁻^1^, and a relative humidity of 65%. Three weeks after planting, leaf tissue from the first true leaves of one plant per genotype was collected for RNA extraction.

### Sampling and RNA extraction

Leaf tissue samples of each genotype, collected from three-week-old seedlings, were immediately frozen in liquid nitrogen and stored at −80 °C until RNA extraction. Total RNA extraction from leaf tissue of each genotype was carried out using the RNeasy Plant Mini Kit (#74,904, QIAGEN, Valencia, CA), following the manufacturer's protocol, and treated with DNase using the Ambion Turbo DNA-Free Kit (#AM1907, Thermo Fisher Scientific, MA, United States). The quality and integrity of the extracted RNA samples were assessed using a NanoDrop ND-1000 spectrophotometer (Saveen Werner, Sweden), and agarose gel electrophoresis with A260/280 = 1.8–2.2 and A260/230 ≥ 2.0 were retained. Subsequently, high-quality RNA samples were dispatched to CD Genomics (NY, USA) for RNA-Seq sequencing. Upon arrival at CD Genomics, sample quality and concentration were assessed using the NanoPhotometer spectrophotometer (IMPLEN, CA, USA), and Qubit RNA Assay Kit in Qubit 2.0 Fluorometer (Life Technologies, CA, USA respectively with Qubit concentration ≥ 50 ng/µL**.** The integrity of RNA was also evaluated using the RNA Nano 6000 Assay Kit of the Agilent Bioanalyzer 2100 system (Agilent Technologies, CA, USA) and samples with RIN ≥ 6.5 were retained for library preparation.

### Library construction and RNA sequencing

Stranded cDNA libraries were constructed utilizing the NEBNext Ultra Directional RNA Library Prep Kit (cat#E7420, NEB, UK) following the manufacturer’s protocols, with index codes added to assign sequences to each sample. Library fragments were purified using an AMPure XP system (Beckman Coulter, Beverly, United States) to preferentially select cDNA fragments ranging from 150–200 bp in length. Sequencing adapters were ligated to size-selected fragments, followed by PCR amplification. After purification of the amplified products using the AMPure XP system, library quality was assessed using the Agilent Bioanalyzer 2100 system. libraries were then normalized to equal molar concentrations based on Qubit quantification and Bioanalyzer size profiles. Normalized libraries were pooled in equimolar ratios. Subsequently, the index-coded samples were clustered on a cBot Cluster Generation System using the TruSeq Cluster Kit v3-cBot-HS (Illumina) as per the manufacturer’s protocol. Finally, sequencing was conducted using an Illumina HiSeq 2500 (San Diego, CA, USA) with 2 × 150 bp read length and > 40 million reads, and then paired-end reads were generated.

### Processing and assembly of transcriptome data

The Illumina HiSeq data were processed into sequenced reads through base calling, creating a FASTQ file containing the sequenced reads and quality information for each sample. Qquality control of raw reads was performed using FastQC v0.11.9, and compiled with MultiQC v1.12. then, raw reads underwent initial cleaning by removing adaptors, reads with excessive Ns, and reads with contig shorter than 200 bp (Phred quality scores below 30%) using Trimmomatic v.0.36. The remaining high-quality reads were utilized for downstream analyses. De novo transcript reconstruction was performed using the Trinity software package [39] with default parameters which include a k-mer size of 25, strand-specific assembly, with minimum contig length cutoff of 200 bp and isoforms were collapsed into unigenes based on shared sequence similarity and read support, followed by the removal of contig with lengths less than 200 bp due to a low annotation rate of short contig [100]. The quality of assemblies was further assessed by evaluating contig counts, N50 statistics, and contig length distributions. Assemblies of the individual genotypes were first assessed for quality and completeness using TransRate and BUSCO, and all individual assemblies were subsequently integrated to create a unified, non-redundant reference transcriptome. To achieve this, assemblies from all genotypes were clustered using CD-HIT-EST at 95–98% sequence identity. Subspecies based individual assemblies were also integrated to create a subspecies based transcriptome assemblies. The longest unigene identified through length distribution analysis was selected as the reference sequence for downstream transcriptome analyses. Consequently, merging of all transcripts generates 108,612 unigenes with a G + C content of 44.8% and used as a reference for SNP calling. The quality-trimmed raw reads were deposited in NCBI Sequence Read Archive (SRA) repository, under BioProject accession number PRJNA1279665. Subspecies based pooling of the individual assemblies also generates 73,106 and 79,085 unigenes for *P. sativum* ssp. *sativum* and *P. sativum* ssp. *abyssinicum*, respectively.

### Functional annotation and classification of the transcriptome

To elucidate the functional characteristics of paired-ended unigenes, a BLASTn search was conducted against the NCBI nucleotide sequence database (Nt) with an E-value threshold of 1e −5. Subsequently, these sequences were queried BLASTp search against other five public databases: Non-Redundant Protein (NR) [101], Kyoto Encyclopedia of Genes and Genomes (KEGG) [102], Universal Protein (UniProt) [103], Plant Transcription Factor Database (PlantTFDB v.3.0) [104], Gene Ontology (GO) [105], and Nucleotide (NT) using BLASTx with the 10e-5 significance level using the default Low-complexity filter (ON). Furthermore, all assembled contigs were compared to the non-redundant protein database (nr) within NCBI to identify the primary species contributing to the annotations. These analyses were performed in 2021 using the latest publicly available versions of all databases at that time. Cluster and Principal Component analyses of expressed genes in both subspecies were carried out using R packages.

### Differentially expressed gene analysis

The paired-end sequenced reads of both genotypes were mapped back to the assembled transcriptome using Bowtie 2 alignment software [106]. After the number of mapped clean reads for each unigene was determined and normalized, the expression level of each transcript was estimated using the RSEM (RNA-Seq by Expectation–Maximization) package (RSEM) v.1.2.08 [107], which is a widely used tool for RNA-Seq data quantification. Differential expression genes were evaluated using DESeq2 and edgeR to assess biological variability and statistical robustness, followed by DEGseq for final identification of differentially expressed genes. Then, the expression level of each gene was determined by calculating the fragments per kilobase pair per million reads (FPKM), among libraries using the formula described by [108]. Accordingly, differentially expressed unigenes between *P. sativum* ssp. *sativum* and *P. sativum* ssp*. abyssinicum* identified with a threshold of FPKM > 0.5, false discovery rate (FDR) ≤ 0.01, and those with an absolute value of log_2_FC ≥ 2 were considered significantly differentially expressed genes between these subspecies.

Differentially expressed genes were visualized using a volcano plot, constructed by plotting the FDR (-log_10_) on the y-axis and the expression fold change between the two *P. sativum* subspecies on the x-axis. The regions of interest in the volcano plot are those found towards the top (high statistical significance) and at the extreme left or right (strongly downregulated and upregulated, respectively).

Furthermore, hierarchical cluster analysis of the genes and genotypes of the two subspecies was performed using the R software package heatmaps v.1.0.8 [109]. In addition, Gene Ontology (GO) classifications and KEGG pathway enrichments were performed between upregulated and down-regulated genes. GO functional enrichment analysis of DEGs was carried out using topGO. DEGs were compared to the Kyoto Encyclopedia of Genes and Genomes (KEGG) database based on BLASTX queries. Gene Ontology (GO) enrichment was carried out using either topGO, applying a hypergeometric test (equivalent to Fisher’s exact test) and KEGG pathway enrichment was performed using KOBAS/clusterProfiler based on KEGG Orthology (KO) assignments generated through DIAMOND BLASTx. For both GO and KEGG analyses, Benjamini–Hochberg false discovery rate (FDR) method, and pathways and GO terms with padj < 0.05 were considered significantly enriched. All DEGs were also searched against the plant transcription factors database (PlantTFDB v.3.0) [110] by setting the E-value cutoff = 10e-10, minimum identity = 40, and minimum query coverage = 50% to identify transcription factor genes among them.

### SSR and SNP analysis

A web-based microsatellite identification tool, MISA-web [111], was used to identify simple sequence repeats (SSRs) within the unigene sequences, using the default setting with the minimum number of repeats of 10, 6, 5, 5, 5, and 5 for mono, di, tri, tetra, penta, and hexa-nucleotide repeats, respectively. The unigenes were scanned for both perfect and compound SSRs using this web based tool. SSRs with two or more SSRs of any types separated by about 100 nucleotides were referred to as compound SSRs. To identify SNPs, high-quality clean reads of the genotypes were aligned to the de novo assembled reference transcriptome using BWA v.0.7.4 short-read aligner [112]. Then, sorting, indexing, removing duplicates, and merging the BAM alignment results of each sample were performed using SAMtools v0.1.18 [113] and Picard-tools v1.41 software packages. The Genome Analysis Toolkit (GATK) [114] was used for base-quality score calibration and SNP calling of the merged BAM files. Genotype calling for each sample was performed using standard filtering parameters or variant quality score calibration according to GATK’s Best Practice recommendations [115]. After merging the VCF files of all samples, BCF tools were used to filter the shared SNP loci among all samples [116]. The genetic collection relationship between the genotypes was analyzed using principal coordinate analysis (PCoA) using GenAlex 6.501 [117], and Polymorphism Information Content (PIC) was calculated using PowerMarker. To further investigate genetic relationships among the genotypes, unweighted pair group method with arithmetic mean (UPGMA) cluster analysis was performed by integrating MEGA with PowerMarker, enabling hierarchical clustering based on genetic distances [118].

## Conclusions

The RNA-Seq analysis conducted in this study effectively identified high-quality transcriptomes, SNPs, SSRs, and DEGs between *P. sativum* ssp. *sativum* and *P. sativu* ssp. *abyssinicum*. This study significantly expands genomic resources for field pea, as most unigenes found limited matches to *Pisum* sequences, underscoring the need for further genomic and transcriptomic characterization. Functional annotation revealed DEGs associated with transcription factors, metabolic pathways, and stress responses, highlighting key genetic distinctions between the two subspecies. Future research should focus on tissue-specific transcriptome profiling to elucidate the genetic basis of phenotypic differences and adaptive traits. Further validating the DEGs using Real-Time PCR and SSR genotyping to confirm their expression patterns and ensure the reliability of the genomic data obtained from high-throughput analyses. Such analyses will clarify molecular mechanisms underlying development, stress responses, and agronomic traits, enabling targeted breeding strategies. The SSR and SNP markers developed here provide valuable tools for breeding, conservation, and unlocking the potential of *P. sativum* ssp. *abyssinicum*, ultimately supporting sustainable agriculture.

## Supplementary Information

Supplementary Figure 1.

Supplementary Figure 2.

Supplementary Figure 3.

Supplementary Table 1.

Supplementary Table 2.

Supplementary Table 3.

Supplementary Table 4.

Supplementary Table 5.

Supplementary Table 6.

Supplementary Table 7.

## Data Availability

The datasets generated and/or analysed during the current study are available in the NCBI Sequence Read Archive (SRA) repository, under BioProject accession number PRJNA1279665, accessible at [https://www.ncbi.nlm.nih.gov/Traces/study/?acc=PRJNA1279665] (https://www.ncbi.nlm.nih.gov/Traces/study/?acc=PRJNA1279665)**.** All other data generated or analyzed during this study are also included in the supplementary information files.
